# Die later with ESCRT!

**DOI:** 10.18632/oncotarget.17903

**Published:** 2017-05-17

**Authors:** Wulf Tonnus, Florian Gembardt, Christian Hugo, Andreas Linkermann

**Affiliations:** Department of Internal Medicine III, Division of Nephrology, University Hospital Carl Gustav Carus at the Technische Universität Dresden, Dresden, Germany

**Keywords:** necroptosis, regulated necrosis, ESCRT, RIPK3, RIPK1

The consequences of necroptosis depend on immunomodulatory molecules, the expression of which requires time before the burst of a cell. Gong et al. now provide evidence for ESCRT-III-mediated plasma membrane repair to extend the time to death during necroptosis. Regulated cell death is not restricted to apoptosis, but includes several forms of regulated necrosis. The best characterized signaling pathway of regulated necrosis is necroptosis. Diverse signaling pathways, such as TNFR1-signaling, TLR-signaling and others may result in receptor-interacting protein kinase 3 (RIPK3)-dependent phosphorylation and oligomerization of the pseudokinase mixed-lineage kinase domain-like (MLKL). It has been proposed that pMLKL may form pores in the plasma membrane, but the actual processes following plasma membrane translocation remain unclear [1]. In a recent report published in Cell, Gong et al. discovered the ESCRT-III-machinery as an active counterpart of pMLKL-associated membrane damage [2]. As necroptosis actively shapes the immune response [3], ESCRT-III indirectly controls the immunogenicity of necroptotically dying cells.

Applying a system to artificially dimerize RIPK3 or MLKL, Gong et al. detected cells that rapidly expose phosphatidylserine (PS) at the outer leaflet of the plasma membrane, a cell death feature that had been associated with apoptosis for the last decades. Single cell analysis revealed the shedding of PS-positive “bubbles”. Unlike apoptotic bodies, these “bubbles” did not contain cytosolic remnants but consisted of broken membranes, as they are permeable to 10 kDa dextran-NH2. These bubbles formed at sites of pMLKL-accumulation, quite similar to what had been previously reported about viral budding in dependence of the ESCRT complex machinery [4]. Indeed, the ESCRT-III-protein CHMP4B co-localized with MLKL at the basis of “bubbles”. A previous study demonstrated ESCRT-mediated repair of laser-damaged membranes [4], but a link to necroptosis was not provided.

In line with this, silencing of ESCRT-III-proteins CHMP2A or CHMP4B resulted in spontaneous necroptosis which was prevented by silencing of RIPK3 or MLKL, or by addition of RIPK1-inhibitor Nec-1s. Conversely, silencing of ESCRT-III-proteins CHMP4B, VPS4B, CHMP2A or ESCRT-I-proteins TSG101 or VPS37B sensitized cells to TNF-induced necroptosis even with active caspase-8 which appears to prevent necroptosis independently of this mechanism. Therefore, this study demonstrates the complex interplay between the ESCRT-III machinery and necroptosis execution, prompting the hypothesis of ESCRT-III to delay the time to plasma membrane rupture and release of damage associated molecular patterns (DAMPs). Indeed, even hardly detectable levels of pMLKL were sufficient to induce necroptosis if ESCRT-III was compromised. Along similar lines, active ESCRT-III delayed membrane breakdown resulting in less chemokine production and less efficient cross-priming of T cells as a consequence of a delay in DAMP release.

MLKL was recently demonstrated to trigger processing and release of both anti-inflammatory cytokines, such as IL-33 and CXCL1, as well as the pro-inflammatory cytokine IL-1β [5]. It will be of interest to precisely unravel the pro- and anti-inflammatory components released by necroptotically dying cells, especially in comparison with cellular cytokines released during distinct pathways of regulated necrosis, such as pyroptosis and ferroptosis [6]. It is possible that the difference in immunogenicity explains the evolutionary conservation of several modes of cell death. In this sense, the timing of plasma membrane rupture in relation to cytokine production may become critical. As all necrotic cell death pathways per definitionem result in DAMP release, the additional expression of either pro- or anti-inflammatory cytokines may provide useful information about the immunogenic nature of these pathways.

**Figure 1:**
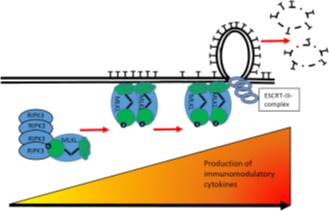
Prevention of necroptosis by ESCRT-III-mediated plasma membrane repair

During necroptosis in kidney transplants, ischemia-reperfusion injury causes formation of the necrosome, a higher order complex that contains RIPK3. This kinase phosphorylates MLKL to signal necroptosis through mechanisms that include translocation to the plasma membrane. Damaged parts of the plasma membrane, identified though of unknown origin, are shed in the forms of bubbles from the plasma membrane to maintain its integrity on the expense of bubbles that are secreted from the cell. This mechanism depends on ESCRT-III and provides time to the dying cell. It is hypothesized that this extension until death is useful for the *de novo* generation of immunomodulatory cytokines, such as CXCL1, IL-33 and IL-1β.

In addition, Gong et al. raise questions about the exact time point, at which a cell must be considered dead. In this study, cells that exposed PS at the outer leaflet - but whose plasma membranes were still intact - could be “resuscitated” by washing out the dimerizing agent or by addition of necrosulfonamide (NSA, a pharmacological inhibitor of MLKL) to human cells.

Apart from ischemic diseases, in which necroptosis contributes to pathophysiology, such as myocardial infarction and acute kidney injury [7], the ESCRT/necroptosis balance may be of particular importance for human transplantation. A role for necroptotic cell death has been previously established [8]. The authors therefore employed a unique set of matched pair transplant biopsies obtained directly before and one hour after living kidney donation. In this human tissue, pMLKL was detected in all samples in the endothelial cells following transplantation, but none of these cells appeared to have lost its plasma membrane integrity. Additionally, the ESCRT-III machinery was significantly upregulated in RNAseq analysis of the transplant samples, providing correlative evidence for the involvement of this system in humans.

In summary, ESCRT-III is an important counterplayer of spontaneous necroptosis, which must be overwhelmed to cause plasma membrane rupture during necroptosis. Therefore, ESCRT-III carries the potential to keep transplanted tissues in a non-immunogenic state without necroptotic DAMP release. It will be interesting to investigate the role of ESCRT-III in cancer cells, which might be overexpressed to keep also cancer cells immunologically silent.
